# Mitochondrial Fission and Mitophagy Coordinately Restrict High Glucose Toxicity in Cardiomyocytes

**DOI:** 10.3389/fphys.2020.604069

**Published:** 2020-12-10

**Authors:** Satoru Kobayashi, Fengyi Zhao, Ziying Zhang, Tamayo Kobayashi, Yuan Huang, Bingyin Shi, Weihua Wu, Qiangrong Liang

**Affiliations:** ^1^Department of Biomedical Sciences, New York Institute of Technology, College of Osteopathic Medicine, Old Westbury, NY, United States; ^2^Department of Endocrinology, The First affiliated Hospital of Xi’an Jiaotong University, Xi’an, China; ^3^Department of Endocrinology, The First Affiliated Hospital of Harbin Medical University, Harbin, China

**Keywords:** diabetes, hyperglycemia, cardiomyocytes, mitochondrial fission, mitophagy, Parkin, DRP1, cell death

## Abstract

Hyperglycemia-induced mitochondrial dysfunction plays a key role in the pathogenesis of diabetic cardiomyopathy. Injured mitochondrial segments are separated by mitochondrial fission and eliminated by autophagic sequestration and subsequent degradation in the lysosome, a process termed mitophagy. However, it remains poorly understood how high glucose affects the activities of, and the relationship between, mitochondrial fission and mitophagy in cardiomyocytes. In this study, we determined the functional roles of mitochondrial fission and mitophagy in hyperglycemia-induced cardiomyocyte injury. High glucose (30 mM, HG) reduced mitochondrial connectivity and particle size and increased mitochondrial number in neonatal rat ventricular cardiomyocytes, suggesting an enhanced mitochondrial fragmentation. SiRNA knockdown of the pro-fission factor dynamin-related protein 1 (DRP1) restored mitochondrial size but did not affect HG toxicity, and Mdivi-1, a DRP1 inhibitor, even increased HG-induced cardiomyocyte injury, as shown by superoxide production, mitochondrial membrane potential and cell death. However, DRP1 overexpression triggered mitochondrial fragmentation and mitigated HG-induced cardiomyocyte injury, suggesting that the increased mitochondrial fission is beneficial, rather than detrimental, to cardiomyocytes cultured under HG conditions. This is in contrast to the prevailing hypothesis that mitochondrial fragmentation mediates or contributes to HG cardiotoxicity. Meanwhile, HG reduced mitophagy flux as determined by the difference in the levels of mitochondria-associated LC3-II or the numbers of mitophagy foci indicated by the novel dual fluorescent reporter mt-Rosella in the absence and presence of the lysosomal inhibitors. The ability of HG to induce mitochondrial fragmentation and inhibit mitophagy was reproduced in adult mouse cardiomyocytes. Overexpression of Parkin, a positive regulator of mitophagy, or treatment with CCCP, a mitochondrial uncoupler, induced mitophagy and attenuated HG-induced cardiomyocyte death, while Parkin knockdown had opposite effects, suggesting an essential role of mitophagy in cardiomyocyte survival under HG conditions. Strikingly, Parkin overexpression increased mitochondrial fragmentation, while DRP1 overexpression accelerated mitophagy flux, demonstrating a reciprocal activation loop that controls mitochondrial fission and mitophagy. Thus, strategies that promote the mutual positive interaction between mitochondrial fission and mitophagy while simultaneously maintain their levels within the physiological range would be expected to improve mitochondrial health, alleviating hyperglycemic cardiotoxicity.

## Introduction

The hallmark feature of diabetes mellitus is the increased blood glucose or hyperglycemia which is an independent risk factor for heart failure in people with diabetes (Stratton et al., 2000; Iribarren et al., 2001; Poornima et al., 2006; Boudina and Abel, 2007). The cellular and molecular mechanisms responsible for hyperglycemia-induced cardiac damage have been extensively studied and multiple hypotheses have been proposed (Jia et al., 2018). A common theory postulates that mitochondrial dysfunction is the major mechanism underlying the pathophysiology of diabetic heart disease (Sivitz and Yorek, 2010; Schilling, 2015). This is supported by both animal and human studies showing the accumulation of damaged mitochondria in the diabetic hearts (Tomita et al., 1996; Frustaci et al., 2000; Shen et al., 2004, 2005; Boudina and Abel, 2006; Bugger and Abel, 2008; Wang et al., 2015). Injured or otherwise dysfunctional mitochondria produce more reactive oxygen species (ROS) and leak out various pro-death factors such as cytochrome C, apoptosis-inducing factor, and Smac/DIABLO (Frustaci et al., 2000; Cai et al., 2002; Ghosh et al., 2005; Malhotra et al., 2005; Shen et al., 2006). Thus, to repair or eliminate injured mitochondrial would be expected to protect against diabetic heart injury.

Mitochondrial quality is controlled by a number of coordinated mechanisms including mitochondrial biogenesis, fission and mitophagy. Dysfunctional mitochondria can be separated by mitochondrial fission and eliminated by autophagic sequestration and subsequent degradation in the lysosome, a process termed mitophagy. Mitochondrial fission is regulated by a number of factors including dynamin-related protein 1 (DRP1), mitochondrial fission protein 1 (Fis1) and mitochondrial fission factor (MFF) (Loson et al., 2013). Fission events generate fragmented mitochondria which is conducive to the sequestration of injured fragments for subsequent degradation through mitophagy. Interestingly, high glucose is able to induce mitochondrial fragmentation in both H9c2 cells (Yu et al., 2006, 2008, 2011) and neonatal cardiomyocytes (Yu et al., 2008, 2017; Makino et al., 2011; Gawlowski et al., 2012) as shown by increased formation of short and small mitochondria. Inhibition of mitochondrial fission by mitochondrial division inhibitor 1 (Mdivi-1) (Yu et al., 2017) and a dominant negative Drp1-K38A mutant (Yu et al., 2008) or increased expression of mitochondrial fusion protein optic atrophy 1 (Opa1) (Makino et al., 2011) attenuates high glucose-induced ROS production and cell death, suggesting that high glucose-induced mitochondrial fragmentation is detrimental. However, blocking mitochondrial fragmentation by Drp1-K38A also inhibits mitochondrial respiration (Yu et al., 2006). The mitochondrial uncoupler FCCP (p-trifluoromethoxy carbonyl cyanide phenyl hydrazone) can induce mitochondrial fragmentation and at the same time reduce high glucose-triggered cardiomyocyte injury (Yu et al., 2006, 2008), suggesting that mitochondrial fragmentation is actually protective under high glucose conditions. Collectively, these opposing results underscore the need of further studies to clarify the true role of mitochondrial fission in hyperglycemic cardiotoxicity.

A well-defined pathway composed of PTEN-induced kinase 1 (PINK1) and Parkin regulates the initiation of mitophagy. Parkin is an E3 ubiquitin ligase that decorates damaged or otherwise depolarized mitochondria for degradation and recycling in the lysosome. Mitophagy is also regulated by a number of adaptors or receptors that are found in cytosol or on mitochondrial membranes (Liang and Kobayashi, 2016). Mitophagy is generally considered to play protective roles in the heart although excessive mitophagy can be detrimental under certain conditions (Song et al., 2015a; Nah et al., 2017). The protein levels of PINK1 and Parkin are decreased in the hearts of both type 1 and 2 diabetic animals (Xu et al., 2013; Tang et al., 2015; Shao et al., 2020), suggesting that mitophagy may be inhibited in the diabetic hearts. Using a novel dual fluorescent mitophagy reporter termed mt-Rosella, we labeled and traced mitochondrial fragments that are sequestered by the autophagosome and delivered to and degraded in the lysosome (Catanzaro et al., 2019; Kobayashi et al., 2020a). We found that mitophagic flux was indeed reduced in high glucose-treated cardiomyocytes and in the heart of streptozocin (STZ)-induced type 1 diabetic mice (Kobayashi et al., 2020a). Deletion of Parkin exacerbated diabetic cardiac injury in mice treated with STZ (Wang et al., 2019) or fed a high fat diet (Tong et al., 2019), suggesting that restoring Parkin-dependent mitophagy may be cardioprotective in diabetes.

Although mitochondrial fission is believed to segregate damaged mitochondria and facilitate their removal by mitophagy (Twig et al., 2008), the functional significance of mitochondrial fission in hyperglycemic cardiotoxicity remains debatable. It is even more controversial how mitochondrial fission couples with and affects mitophagy activity in the diabetic heart (Liang and Kobayashi, 2016). In the present study, we determined the activities of, and the interaction between, mitochondrial fission and mitophagy in high glucose-treated cardiomyocytes. Our results demonstrated that high glucose elicited coordinated changes in the activities of mitochondrial fission and mitophagy, which collectively limit high glucose toxicity.

## Materials and Methods

### Neonatal Rat Ventricular Cardiomyocyte Culture and High Glucose and/or Drug Treatments

We prepared neonatal rat ventricular cardiomyocytes (NRVC) from neonatal Harlan Sprague-Dawley rats and cultured them in Dulbecco’s Modified Eagle Medium (DMEM, GIBCO, 11966) as described previously (Kobayashi et al., 2012). These NRVCs were cultured for 12–72 h in glucose-free DMEM (Gibco 11966025, Thermo Fisher Scientific) supplemented with 5.5 or 30 mmol/liter of D-(+)-glucose (Sigma, G7021). For some experiments, NRVCs were treated with lysosomal inhibitors Pepstatin A (PepA) (12.5 μg/mL, RPI, P30110, Mount Prospect, IL, United States) and E64d (5 μg/mL, RPI, E57050, Mount Prospect, IL, United States), mdivi-1 (1 uM, Sigma, M0199) and mitochondrial uncoupler Carbonyl cyanide m-chlorophenylhydrazone (CCCP) (5 nM, Sigma, C2759). The osmolarities of all media were adjusted to 30 mM by mannitol (Sigma, M9647). We added 100 units/ml penicillin and streptomycin (Sigma, P4333) to all media.

### Replication-Deficient Adenoviruses

We purchased the human DRP1 and Parkin cDNA clones from OriGene and obtained the mitophagy reporter mt-Rosella from Dr. Devenish (Mijaljica et al., 2011). Rosella is a fusion protein that contains a mitochondrial targeting sequence, a pH-stable red fluorescent protein (RFP) and a pH-sensitive green fluorescent protein (GFP). The replication-deficient adenoviral vectors expressing DRP1, Parkin, or mt-Rosella were generated using the AdEasy Adenoviral Vector System (Stratagene, 240009) as we described previously (Catanzaro et al., 2019). Unless otherwise indicated, we infected NRVCs with adenovirus at a multiplicity of infection (MOI) of 100 plaque forming unit (pfu) for 24 h before drug treatment.

#### Confocal Microscopy and Mitophagy Analysis

Ad-mt-Rosella-infected NRVC were fixed with 4% paraformaldehyde prepared in PBS for 15 min at room temperature. Dual-fluorescent images of NRVC infected with Ad-mt-Rosella were obtained using Nikon C2 (Nikon) confocal microscope at 60× magnification or LSM900 with Airyscan 2 (Zeiss) confocal microscope at 63× magnification (1.4 numerical aperture). The GFP quenched “red only” mitophagy foci were isolated by subtracting the green channel from the red channel using ImageJ’s Image Calculator after splitting two color channels. After the image optimization, ImageJ’s particle analysis was performed to obtain the number of mitophagy foci with a size threshold of 5 to 50px^2^ in order to exclude background noises and large aggregates or nuclei, which were unlikely to represent mitophagy foci. At least five images (each containing between 5 and 8 cells) were captured and analyzed per treatment. For determining mitophagy flux, experiments were duplicated with addition of lysosomal inhibitors PepA (12.5 μg/mL) and E64d (5 μg/mL). Mitophagy flux was calculated by subtracting the mean of red puncta without inhibitors from the corresponding mean value of red puncta with inhibitors.

### Mitochondrial Morphology Analysis

For live cell imaging, cultured NRVCs were stained with 20 nM of MitoTracker Green FM (M7514, Thermo Fisher Scientific, Waltham, MA, United States) for 30 min in CO^2^ incubator at 5% of CO^2^ and 37°C. The cells infected with adenovirus encoding mt-Rosella reporter were fixed with 4% paraformaldehyde. The live cells were imaged at 100× magnification (1.4 numerical aperture) with Fluoview FV1000 (Olympus) and the fixed cells were imaged by using Nikon C2 (Nikon) confocal microscope at 60× magnification (1.4 numerical aperture), using the linear sequential scan mode (excitation/emission filter, 488/510 nm; 561/592 nm), 1072 × 1072 resolution. The images were processed with Fiji/ImageJ. Raw images were contrast enhanced and then binarized to apply a threshold to highlight mitochondrial structures. After optimization, the relative mitochondrial sizes, numbers and relative mass in each cell were obtained by particle analysis measurements. At least five images (each containing between 5 and 8 cells) were captured and analyzed per treatment.

#### Mitochondrial Superoxide Measurement (MitoSOX Assay)

Prior to imaging, the cells were incubated with 5 μM MitoSOX (M36008, Invitrogen, Thermo Fisher Scientific, Waltham, MA, United States) for 10 min at 37°C. The cells were examined using a fluorescent microscope to detect and image the fluorescent signals. Three to Five images were obtained from each treatment group. The images were analyzed by ImageJ to quantify the red fluorescence intensity of each treatment.

#### Mitochondrial Membrane Potential Measurement (ΔΨm)

MitoPT JC-1 Assay Kit (924, ImmunoChemistry, Bloomington, MN, United States) was used for assessing mitochondrial membrane potential (ΔΨm). The cultured cells were incubated with JC-1 dye for 30 min and then analyzed by Cytation fluorescence plate reader (BioTek), with excitation at 488 nm and emission at 590 nm (aggregates) and at 527 nm (monomeric). The stained images were separately obtained by Fluorescence microscopy (IX71 Olympus) at 10× magnification.

### Western Blot Analysis

Cultured NRVCs were processed for Western blot analysis as described previously (Xu et al., 2012, 2013). Briefly, NRVCs were collected in 1 × SDS, boiled for 10 min, loaded to polyacrylamide gel for electrophoresis, and then transferred to polyvinylidene difluoride membranes. The membranes were incubated with primary and secondary antibodies, and processed for chemiluminescent detection using Lumigen ECL Ultra (TMA-6 Lumigen, Southfield, MI, United States). The images were acquired by using Amersham Imager 600 and quantified with ImageJ. The antibodies against cleaved Caspase 3 (cCasp3) (#9664), β-Actin (#4967), microtubule-associated protein Light Chain 3 (LC3B, #3868), Parkin (#2132), glyceraldehyde-3-phosphate dehydrogenase (GAPDH) (#5147), Voltage Dependent Anion Channel 1 (VDAC1) (#4866), Peroxisome proliferator-activated receptor gamma coactivator 1-alpha (PGC-1α) (#4259) and Cytochrome c oxidase subunit 4 (COX IV) (#4850) were purchased from Cell Signaling Technology (Danvers, MA, United States). The OPA1 antibodies (ab42364) were purchased from Abcam (Cambridge, MA, United States). The antibodies against Mfn1 (sc-166644), Mfn2 (sc-100560), PINK1 (sc-33796), and DRP-1 (sc-271583) as well as the horseradish peroxidase-conjugated secondary antibodies (sc-2004, sc-2005, sc-2020, and sc-2438) were obtained from Santa Cruz Biotechnology.

### SiRNA Gene Silencing

We obtained short interference RNAs (siRNAs) targeting DRP1 or Parkin mRNA and a Silencer Negative Control siRNA from Ambion (Austin, TX, United States). The knockdown of DRP1 or Parkin was achieved by transfecting NRVCs with a mixture of three different siRNA oligoes, each at a concentration of 16.67 nM as described previously (Catanzaro et al., 2019; Kobayashi et al., 2020b).

### Cell Death Assays

The dead cells were identified by staining the NRVCs with 2 μg/mL of Propidium iodide (PI) for 10 min and examined under a fluorescent microscope. Three to five images of red fluorescence and phase contrast were obtained from each treatment group. The number of PI positive cells were counted and expressed as a percentage of the total cell number examined per treatment. Apoptotic cell death was determined by Western blot analysis of cCasp3 and cleaved PARP, as described above.

### Preparation and Culture of Adult Mouse Cardiomyocytes

Adult mouse cardiomyocytes (AMCs) were isolated from 8-week-old transgenic mice that express the mitophagy reporter mt-Rosella in the heart (Kobayashi et al., 2020a) and cultured using the method described by Ackers-Johnson (Ackers-Johnson et al., 2016). Briefly, the heart was removed and perfused with collagenase solution by slow injection of the buffer into the left ventricle with a syringe. The isolated AMCs were cultured in M199 media containing the designated concentrations of glucose for 72 h and then fixed with 2% paraformaldehyde in culture media for 15 min for confocal imaging.

### Determination of Mitochondrial DNA (mtDNA) Copy Number

Total DNA was extracted from 1.0 × 10^6^ cultured NRVCs by using the Phenol-Chloroform method. Cells were lysed with Phenol:Chloroform:Isoamyl Alcohol (25:24:1), saturated with 10 mM Tris, pH 8.0, 1 mM EDTA (Sigma P3803). Extracted DNA was dissolved in Nuclease free water and quantified by using the NanoDrop ND-1000 Spectrophotometer (Labtech International Ltd.). Quantitative PCR analysis was performed using the SYBR Green PCR Master Mix (Thermo Fisher Scientific, Applied Biosystems, #4364344) on the Applied Biosystems StepOne Real-Time PCR Systems (Applied Biosystems) with 20 uL of reaction mixture containing 50 ng/uL of DNA and 200 nM of primers. The oligonucleotide primers used for reaction are as follows: COX IV gene for mitochondrial DNA, forward: 5′-CCCCTGCTATAACCCAATACA-3′, backward: 5′-CCAAACCCTGGAAGAATTAAGA-3′; GAPDH for nucleic DNA, forward: 5′- TGTTGCTGTAGCCATATTCATTGT-3′, backward: 5′- CCATTCTTCCACCTTTGATGCT -3′.

### Statistical Analysis

Data were expressed as the mean ± SEM. Unpaired Student *t*-test and one-way or two-way analysis of variance (ANOVA) were used to analyze the differences between experimental groups followed by Tukey’s Multiple Comparison Test using GraphPad Prism Version 8. *p* < 0.05 was considered statistically significant.

## Results

### High Glucose Induced Mitochondrial Fragmentation in NRVCs

**FIGURE 1:**
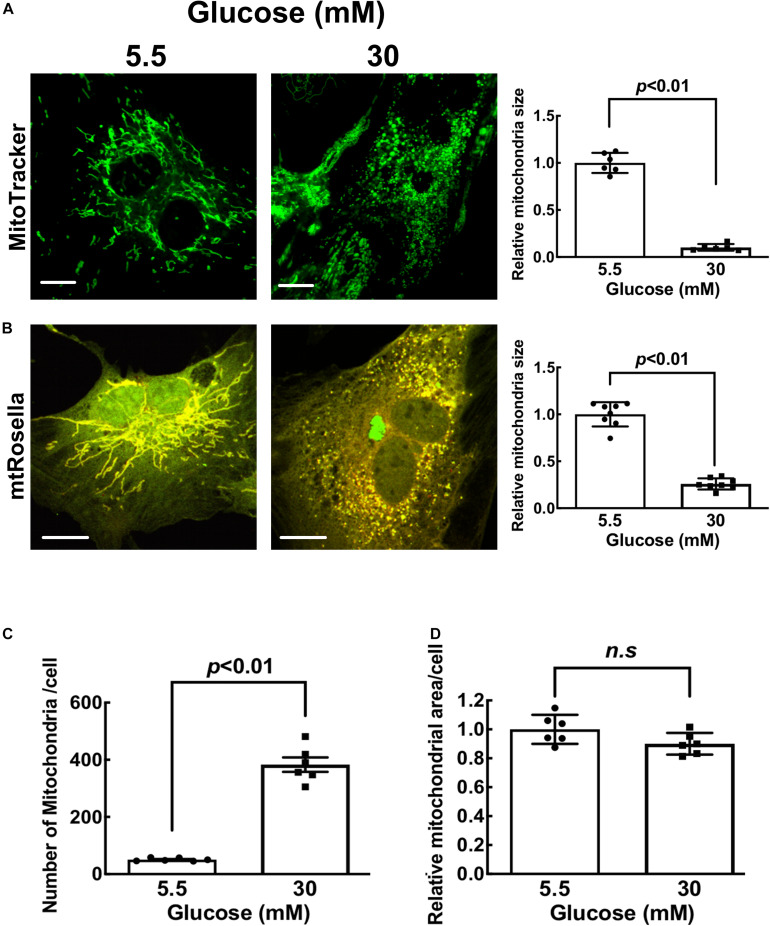
High glucose induces mitochondrial fragmentation in cardiomyocytes. NRVCs were cultured in DMEM with 5.5 or 30 mM glucose for 72 h. Mitochondrial structure was visualized by staining with MitoTracker Green (live cells, panel **A**) or dual fluorescence mt-Rosella mitophagy reporter (fixed cells, panel **B**). The confocal images of MitoTracker and mt-Rosella were analyzed with ImageJ. After image optimization, mitochondrial morphology was analyzed and average particle size **(A,B)**, mitochondrial numbers **(C)** and total areas **(D)** were quantified. At least five images (each containing 5–8 cells) were captured per treatment. The scale bars = 10 μm **(A)** and 20 μm **(B)**. Data are expressed as mean ± SEM and analyzed by using unpaired student *t*-test (^∗^*p* < 0.01 vs. 5.5 mM glucose, *n* = 6–8).

To characterize the effects of high glucose on mitochondrial morphology in cardiomyocytes, NRVCs were cultured with 5.5 mM [normal glucose (NG)] or 30 mM [high glucose, (HG)] for 3 days and then stained with MitoTracker Green for live imaging. Mitochondria in NG-cultured cells appeared elongated and connected. In contrast, mitochondria in HG-cultured cells were smaller and separated, suggesting that HG induces mitochondrial fragmentation (Figure 1A). Indeed, the mean mitochondrial particle size was substantially reduced by HG treatment (*p* < 0.01, *n* = 6). Alternatively, NRVCs were infected with adenovirus encoding the dual fluorescent mt-Rosella reporter which stains mitochondria and tracks their whereabouts in the cell as described below and previously (Catanzaro et al., 2019; Kobayashi et al., 2020a). These cells were fixed with 4% paraformaldehyde and imaged. The merged confocal images showed yellow mitochondria which appeared more fragmented in HG-cultured cells compared with NG as indicated by the reduced mitochondrial size (*p* < 0.01, *n* = 8, Figure 1B) and increased the mitochondrial number (Figure 1C). These results replicated those from live cells stained with MitoTracker Green, reinforcing the notion that HG is able to induce mitochondrial fragmentation. We also performed a time-course study and found that mitochondrial fragmentation started as early as 12 h after HG treatment and plateaued 72 h later as shown by the reduced mitochondrial size and increased mitochondrial number (Supplementary Figure 1). Interestingly, HG had no effects on the total mitochondrial area (Figure 1D), suggesting that HG might have not affected mitochondrial degradation. To explore the potential mechanisms responsible for the increased fission, we screened several common regulators of mitochondrial fission and fusion. Although previous studies reported increased levels of Drp1 protein or its phosphorylation by HG (Yu et al., 2017), we did not reproduce these results. Instead, we found that HG treatment decreased the fusion protein mitofusin 1 (Mfn1) in mitochondria fractions (Supplementary Figure 2A), suggesting a reduced mitochondrial fusion. Mitochondrial morphology is determined by the balance between mitochondrial fusion and fission. The reduced Mfn1 likely tilted the balance in favor of fission, leading to an increased mitochondrial fragmentation.

### Moderate DRP1 Knockdown Did Not Affect High Glucose-Induced Cardiomyocyte Injury

**FIGURE 2:**
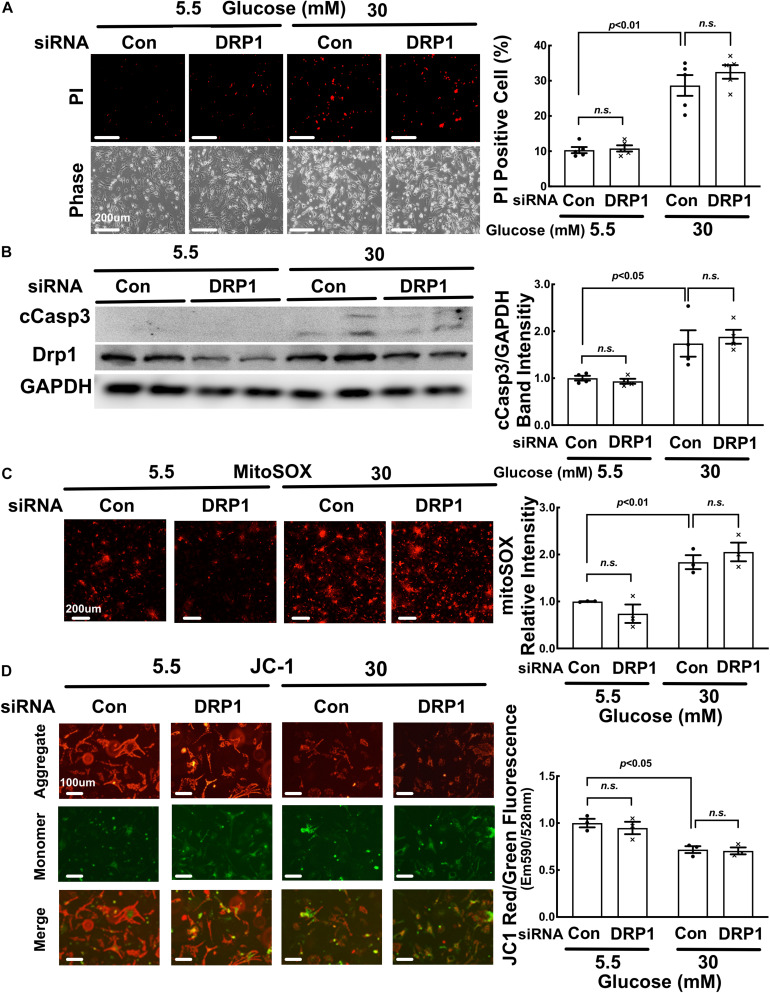
Moderate DRP1 knockdown did not affect high glucose-induced cardiomyocyte injury. NRVCs were transfected with scrambled control or DRP1-targeted synthetic siRNA, cultured with DMEM containing 5.5 or 30 mM glucose for 72 h, and then cardiomyocyte injury was determined by the number of PI positive cells (**A:** *p* > 0.05, *n* = 5), the levels of cCasp3 (**B:** cCasp3, *p* > 0.05, *n* = 4), superoxide production (**C:** MitoSOX, *p* > 0.05, *n* = 3–4) and mitochondrial membrane potential (**D:** ΔΨm, JC-1, *p* > 0.05, *n* = 3). Data were expressed as mean ± SEM and analyzed by two-way ANOVA. Scale bars represent **(A)** 200 μm, **(C)** 200 μm, **(D)** 100 μm, respectively.

**FIGURE 3:**
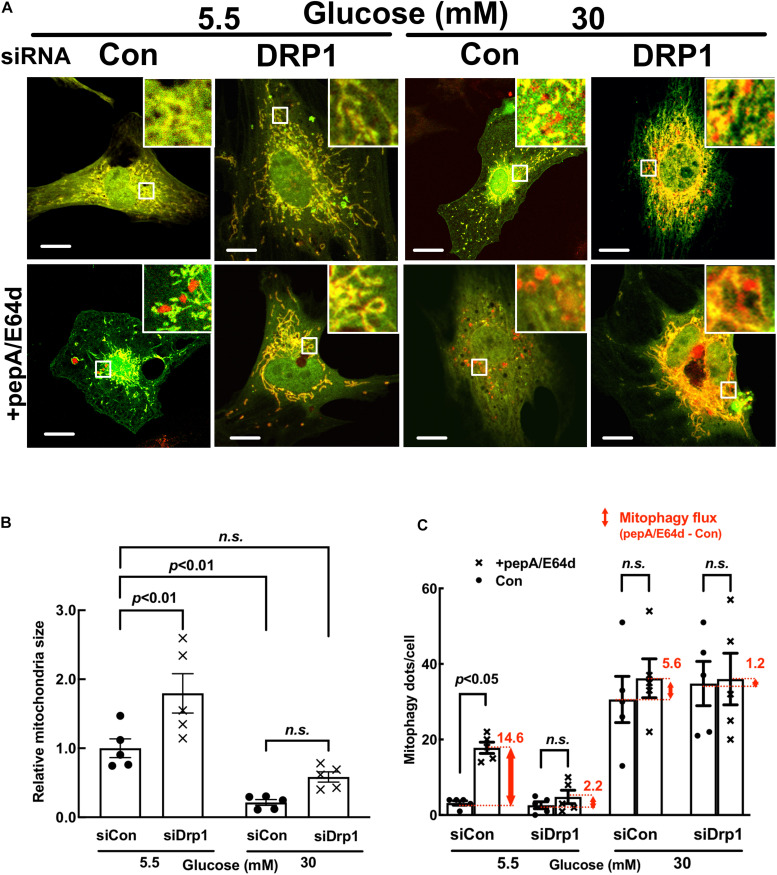
DRP1 knockdown reduced basal mitochondrial fission and mitophagy flux in cardiomyocytes. NRVCs were infected with Admt-Rosella, transfected with scrambled control or DRP1-targeted synthetic siRNA, and then cultured with DMEM containing 5.5 or 30 mM glucose for 72 h. Mitochondrial morphology was observed with confocal microscopy and the merged confocal images **(A)** were analyzed using ImageJ. The relative mitochondrial sizes **(B)** and the number of mitophagy foci (red puncta or dots, **C**) in each cell were obtained by particle analysis measurements. At least five images (each containing between 5 and 8 cells) were captured per treatment. Scale bar = 20 μm. Experiments were repeated with addition of lysosomal inhibitors (PepA and E64D). Mitophagy flux was calculated by subtracting the mean of red puncta without inhibitors from the corresponding mean value of red puncta with inhibitors, which was denoted by the red number and the red vertical line with arrows at both ends in the bar graphs. Data were expressed as mean ± SEM and analyzed by two-way ANOVA (*n* = 5 for each group with *p* values indicated in the graphs).

To determine the functional role of fission in high glucose (HG) toxicity in NRVCs, we used synthetic siRNAs to knock down the protein expression of DRP1, a major factor that promotes mitochondrial fission. Since complete DRP1 knockdown caused cell death, we kept the knockdown at a moderate level (∼50%, Figure 2B). As shown in Admt-Rosella-infected NRVCs (Figure 3A) and the quantification (Figure 3B), moderate DRP1 knockdown increased the mean mitochondrial particle size in NG-cultured NRVCs (*p* < 0.01, *n* = 5), confirming the ability of siDRP1 to reduce baseline mitochondrial fission. SiDRP1 also showed a trend to attenuate HG-induced fragmentation (*p* = NS, *n* = 5). However, moderate DRP1 knockdown did not impact either the viability of NRVCs under NG conditions or HG-induced cardiomyocyte injury, as shown in Figure 2 by the number of PI positive cells (Figure 2A: *p* > 0.05, *n* = 5), the levels of cCasp3 (Figure 2B: cCasp3, *p* > 0.05, *n* = 4), superoxide production (Figure 2C: MitoSOX, *p* > 0.05, *n* = 3–4) and mitochondrial membrane potential (Figure 2D: ΔΨm, JC-1, *p* > 0.05, *n* = 3). More surprisingly, inhibiting DRP1-dependent mitochondrial fission with mdivi-1 exacerbated HG-induced cardiomyocyte death as shown by the increased PI positive cells and cCasp3 (Supplementary Figures 3A,B). These results suggested that reducing mitochondrial fragmentation *per se* did not prevent or protect from HG cardiotoxicity; rather, it increased HG cardiotoxicity, in contrast to a previous study showing that inhibition of mitochondrial fission attenuated HG-induced cardiomyocyte death (Yu et al., 2008).

### DRP1 Overexpression Triggered Mitochondrial Fission and Protected From HG-Induced Cardiomyocyte Injury

**FIGURE 4:**
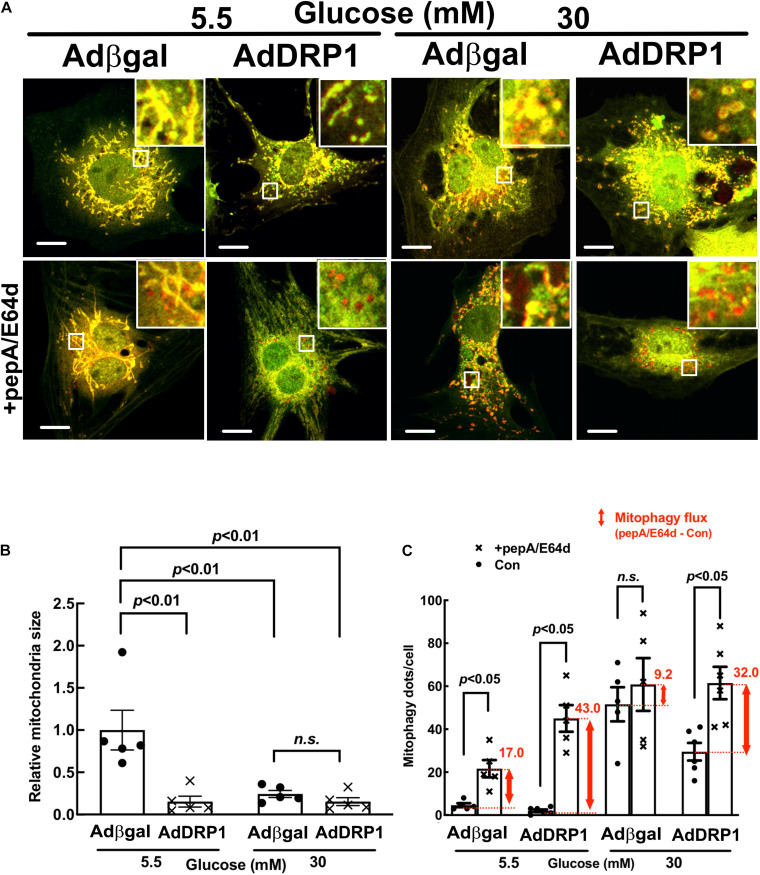
DRP1 overexpression triggered mitochondrial fission, increased basal mitophagy and alleviated the inhibition of mitophagy flux by high glucose (HG). NRVCs were infected with AdDRP1 or Adβgal and cultured with DMEM containing 5.5 or 30 mM glucose for 72 h. Mitochondrial morphology was examined with confocal microscopy and the merged confocal images **(A)** were analyzed using ImageJ. The relative mitochondrial sizes **(B)** and the number of mitophagy foci (red puncta or dots, **C**) in each cell were obtained by particle analysis measurements. At least five images (each containing between 5 and 8 cells) were captured per treatment. Scale bar = 20 μm. Mitophagy flux was measured with and without PepA and E64D, and denoted by the red number and the red vertical line with arrows at both ends in the bar graphs. Data were expressed as mean ± SEM and analyzed by two-way ANOVA (*n* = 5 for each group with *p* values indicated in the graphs).

**FIGURE 5:**
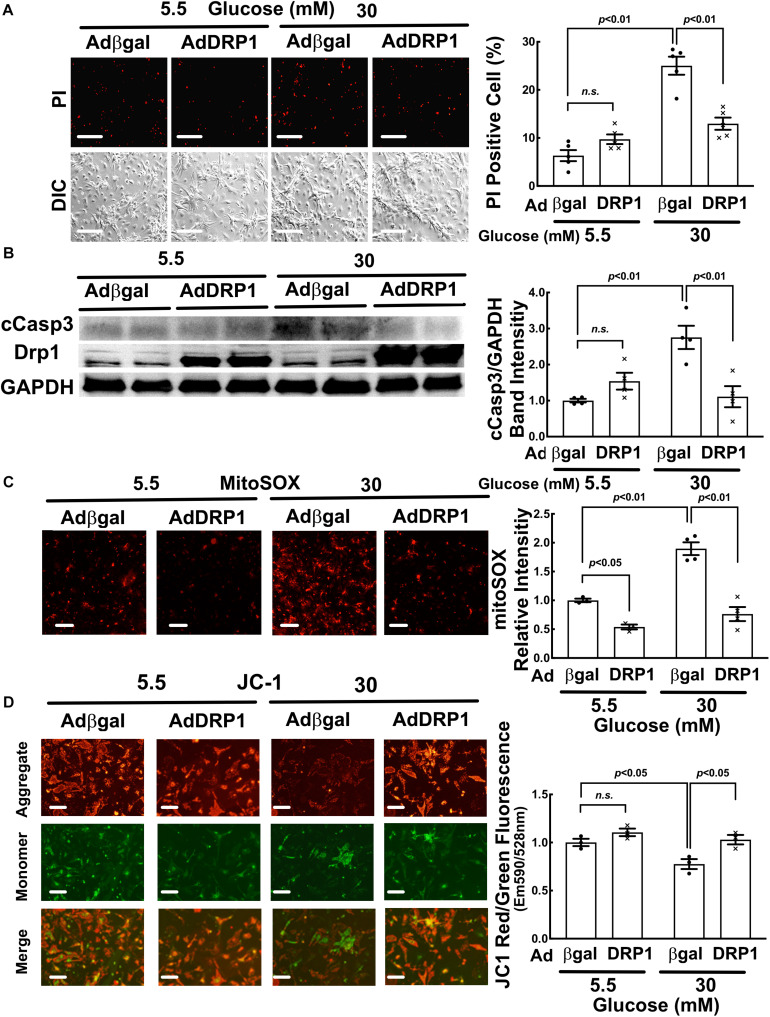
DRP1 overexpression alleviated high glucose-induced cardiomyocytes injury. NRVCs were infected with AdDRP1 or Adβgal and cultured in DMEM containing 5.5 or 30 mM glucose for 72 h, and then cardiomyocyte injury was determined by the number of PI positive cells (**A:** *p* < 0.01, *n* = 5), the levels of cCasp3 (**B:** *p* < 0.01, *n* = 4), MitoSOX (**C:** *p* < 0.01, *n* = 3) and ΔΨm (**D:** JC-1, *p* < 0.05, *n* = 3). Data were expressed as mean ± SEM and analyzed by two-way ANOVA. Scale bars represent **(A)** 200 μm, **(C)** 200 μm, **(D)** 100 μm, respectively.

We used adenovirus-mediated gene transfer to determine if DRP1 overexpression could trigger mitochondrial fission in NRVCs. As expected, DRP1 overexpression (AdDRP1) induced mitochondrial fragmentation at NG as shown by the smaller and less connected mitochondria (Figures 4A,B, *p* < 0.01, *n* = 5), but it failed to further increase HG-induced mitochondrial fragmentation. In addition, whereas DRP1 overexpression only reduced the production of mitochondrial superoxide (MitoSOX) under NG conditions, it surprisingly mitigated HG-induced cardiomyocyte injury as shown by PI positive cells (Figure 5A, *p* < 0.01, *n* = 5), cCasp 3 (Figure 5B, *p* < 0.01, *n* = 4), superoxide (Figure 5C, *p* < 0.01, *n* = 4) and ΔΨm (Figure 5D, *p* < 0.05, *n* = 3). These results demonstrated that increasing mitochondrial fragmentation did not contribute to HG cardiotoxicity; instead, it protected against HG-induced cardiomyocyte injury, suggesting that mitochondrial fragmentation was actually an adaptive response that limited HG toxicity, in stark contrast to the study reporting mitochondrial fission as a mediator of HG toxicity (Yu et al., 2008).

### High Glucose (HG) Inhibited Mitophagy Flux in NRVCs

**FIGURE 6:**
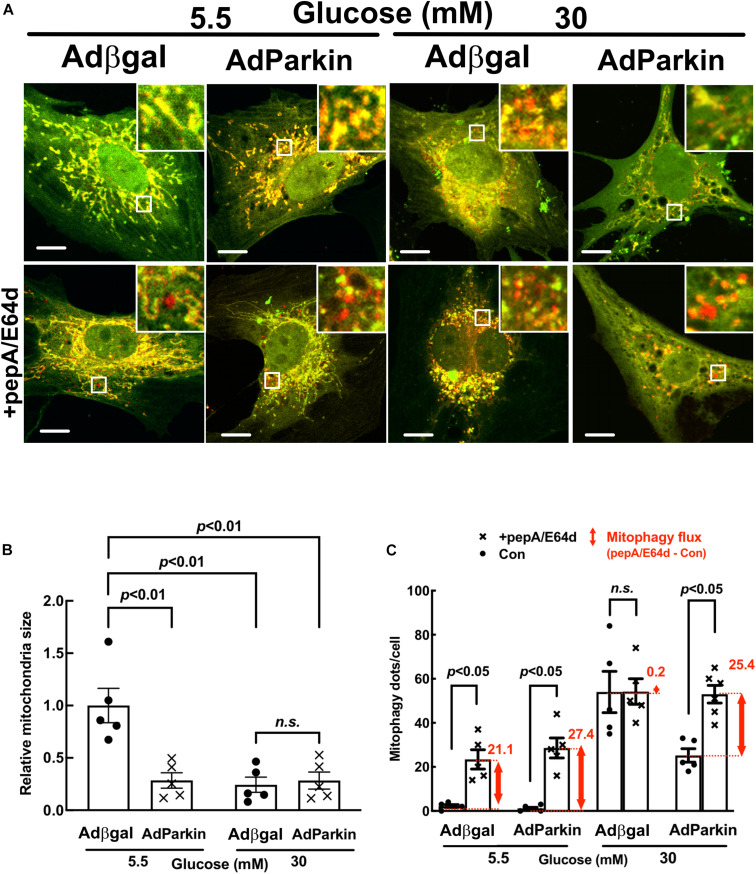
Parkin overexpression increased basal mitophagy and relieved the inhibition of mitophagy flux by high glucose. NRVCs were infected with Admt-Rosella and AdParkin or Adβgal and cultured in DMEM containing 5.5 or 30 mM glucose for 72 h. Mitochondrial morphology was examined with confocal microscopy and the merged confocal images **(A)** were analyzed using ImageJ. The relative mitochondrial sizes **(B)** and the number of mitophagy foci (red puncta or dots, **C**) in each cell were obtained by particle analysis measurements. At least five images (each containing between 5 and 8 cells) were captured per treatment. Scale bar = 20 μm. Mitophagy flux was measured with and without PepA and E64D, and denoted by the red number and the red vertical line with arrows at both ends in the bar graphs. Data were expressed as mean ± SEM and analyzed by two-way ANOVA (*n* = 5 for each group with *p* values indicated in the graphs).

**FIGURE 7:**
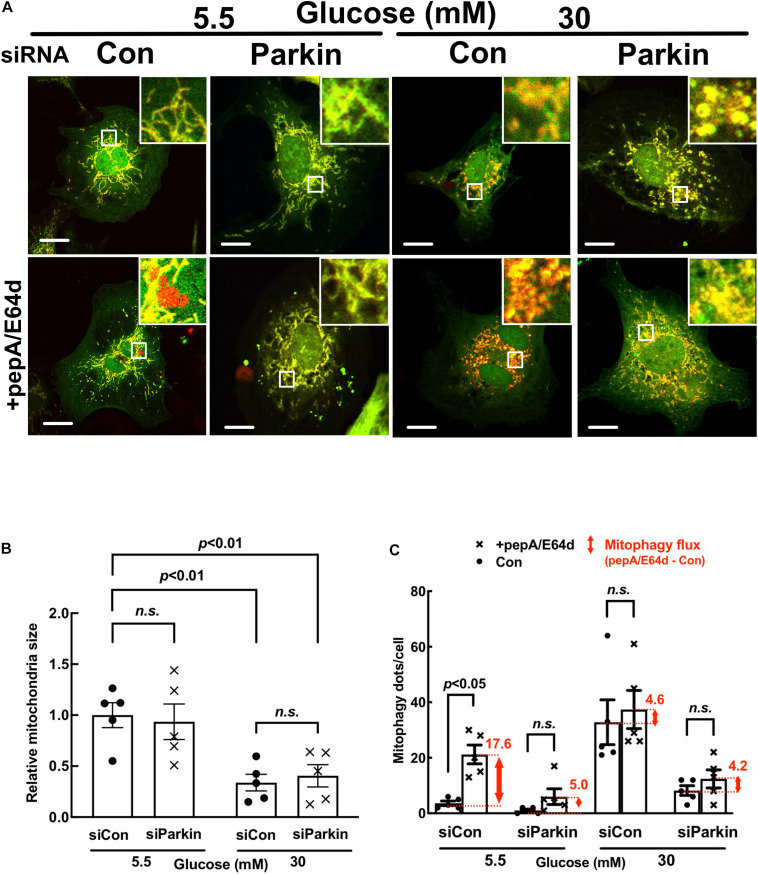
Parkin knockdown inhibited mitophagy flux in cardiomyocytes. NRVCs were infected with Admt-Rosella, transfected with scrambled control or Parkin-targeted synthetic siRNA, and cultured with DMEM containing 5.5 or 30 mM glucose for 72 h. Mitochondrial morphology was examined with confocal microscopy and the merged confocal images **(A)** were analyzed using ImageJ. The relative mitochondrial sizes **(B)** and the number of mitophagy foci **(C)** in each cell were obtained by particle analysis measurements. At least five images (each containing between 5 and 8 cells) were captured per treatment. Scale bar = 20 μm. Experiments were repeated with addition of lysosomal inhibitors (PepA and E64D) for evaluating mitophagy flux which was denoted by the red number and the red vertical line with arrows at both ends in the bar graphs. Data were expressed as mean ± SEM and analyzed by two-way ANOVA (*n* = 5 for each group with *p* values indicated in the graphs).

**FIGURE 8:**
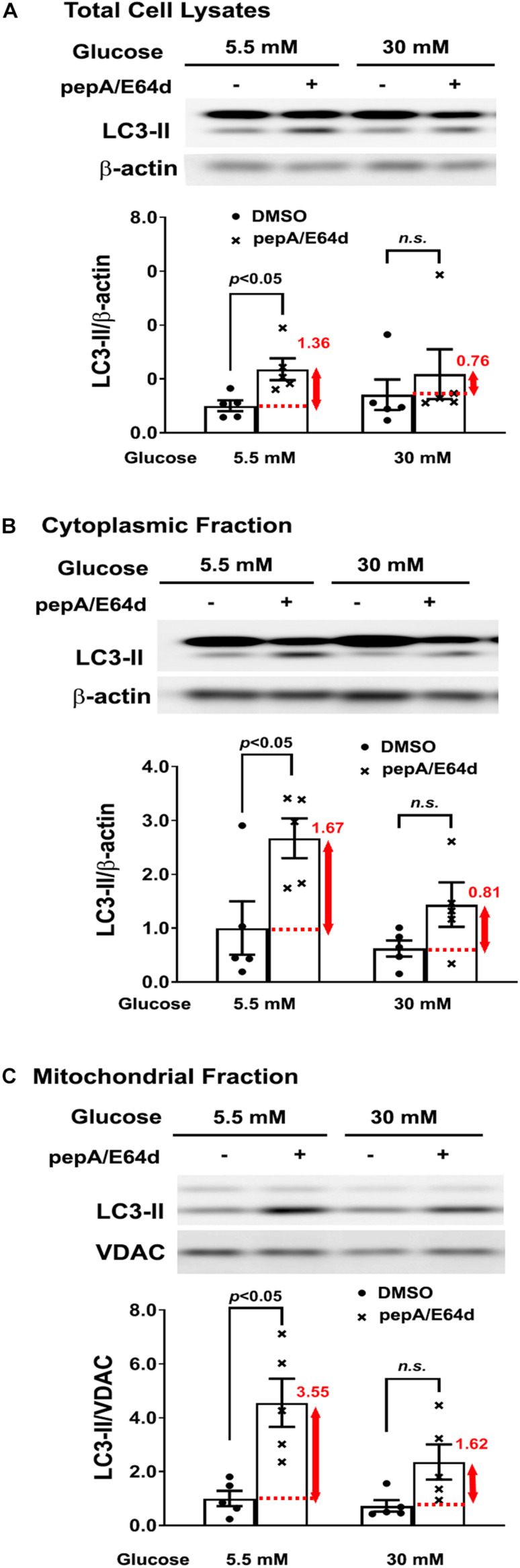
High glucose inhibited mitophagy flux. NRVCs were cultured in DMEM containing 5.5 or 30 mM glucose for 72 h. The LC3-II levels were determined by Western blot analysis in total cell lysates **(A)**, cytoplasmic fractions **(B)** or mitochondrial fractions **(C)** with or without adding PepA and E64d. Mitophagy flux was denoted by the red number and the red vertical line with arrows at both ends in the bar graphs. Data were expressed as mean ± SEM and analyzed by two-way ANOVA (*n* = 5 for each group with *p* values indicated in the graphs).

To directly visualize and quantify the mitochondrial fragments that are degraded through the mitophagic process, we constructed an adenovirus that encodes mt-Rosella, a novel mitophagy reporter (Catanzaro et al., 2019; Kobayashi et al., 2020a) that is a mitochondria-targeted dual-emission biosensor containing RFP and GFP as a fusion protein (Mijaljica et al., 2011). We infected NRVCs with Ad-mt-Rosella and compared mitophagy events (red fluorescent dots or puncta on the merged confocal images) in cells cultured under HG (30 mM) and NG (5.5 mM) conditions. As shown by the control groups in Figures 3A,C (siRNA control), Figures 4A,C (Adβgal), Figures 6A,C (Adβgal) and Figures 7A,C (siRNA control), HG increased the number of red dots compared with NG, suggesting that HG might have enhanced mitophagy. However, treatment with lysosomal degradation inhibitors PepA and E64d resulted in a smaller increase in the number of red dots in cells cultured in HG than in NG, suggesting that HG actually reduced the mitochondria degradation rate in the lysosome. In other words, HG inhibited mitophagy flux, consistent with our previous results (Kobayashi et al., 2020a). Mitophagy flux reflects the number of mitochondria that are delivered to and degraded in the lysosome. It was determined by the difference in the numbers of mitochondria trapped in the lysosome (red puncta on merged confocal images) before and after applying lysosomal degradation inhibitors. The mitophagy flux was denoted by the red number and the red vertical line with arrows at both ends in each of these bar graphs (Figures 3C, 4C, 6C, 7C). To further characterize mitophagy flux, we determined the effects of HG on the levels of autophagosomal membrane marker LC3-II that were associated with the mitochondrial fraction with and without PepA and E64d treatment. As shown in Figure 8, the LC3-II levels in total cell lysates (Figure 8A), cytoplasmic fractions (Figure 8B) or mitochondrial fractions (Figure 8C) were not significantly different between HG and NG cultures in the absence of PepA and E64d. However, HG caused a considerably less increase in LC3-II levels than NG when PepA and E64d was used to block lysosomal degradation, suggesting that HG inhibited mitophagy flux, consistent with the results from the mt-Rosella mitophagy reporter. Given the reduced mitophagy and the unchanged mitochondrial amount (Figure 1D), it’s possible that HG might have reduced mitochondrial biogenesis, which could helps maintain a relatively normal mitochondrial mass. Indeed, we found that HG reduced mitochondrial DNA content (Supplementary Figure 2B) and had a tendency to decrease the protein levels of PGC-1α (Supplementary Figure 2A), a master regulator of mitochondrial biogenesis. These results support the idea that HG inhibited mitochondrial biogenesis which may explain why mitochondrial mass remained normal despite the reduced mitophagy.

### DRP1 Overexpression Alleviated the Inhibition of Mitophagy Flux by High Glucose (HG)

Mitochondrial fission and mitophagy are two cellular mechanisms that coordinately control mitochondrial quality. Fission process is essential for mitophagy in that it separates injured mitochondrial segments which in turn are sequestered, delivered to and degraded in the lysosome. We determined if adenovirus-mediated gene transfer of DRP1 could affect mitophagy flux in NRVCs using the mt-Rosella reporter. As shown in Figures 4A,C, AdDRP1 infection reduced the number of red dots as compared with Adβgal under both NG and HG conditions, suggesting that HG might have attenuated mitophagy. However, treatment with PepA and E64d led to a larger increase in the number of red dots in cells infected with AdDRP1 than those with Adβgal under either NG or HG conditions. The results suggested that DRP1 not only increased mitophagy flux in NRVCs cultured under NG conditions, but also alleviated the inhibition of mitophagy flux by HG. Thus, DRP1 may reduce HG toxicity through its ability to increase mitophagy flux. We also examined the effects of siRNA-mediated DRP1 knockdown on mitophagy. Although siDRP1 substantially slowed mitophagy flux in NRVCs cultured in NG media, its effect on HG-inhibited mitophagy was relatively small (Figures 3A,C), probably because mitophagy flux was already depressed by HG. This may explain why DRP1 knockdown did not alter HG toxicity (Figure 2).

### Parkin Overexpression Accelerated Mitophagy Flux and Reduced HG Toxicity

**FIGURE 9:**
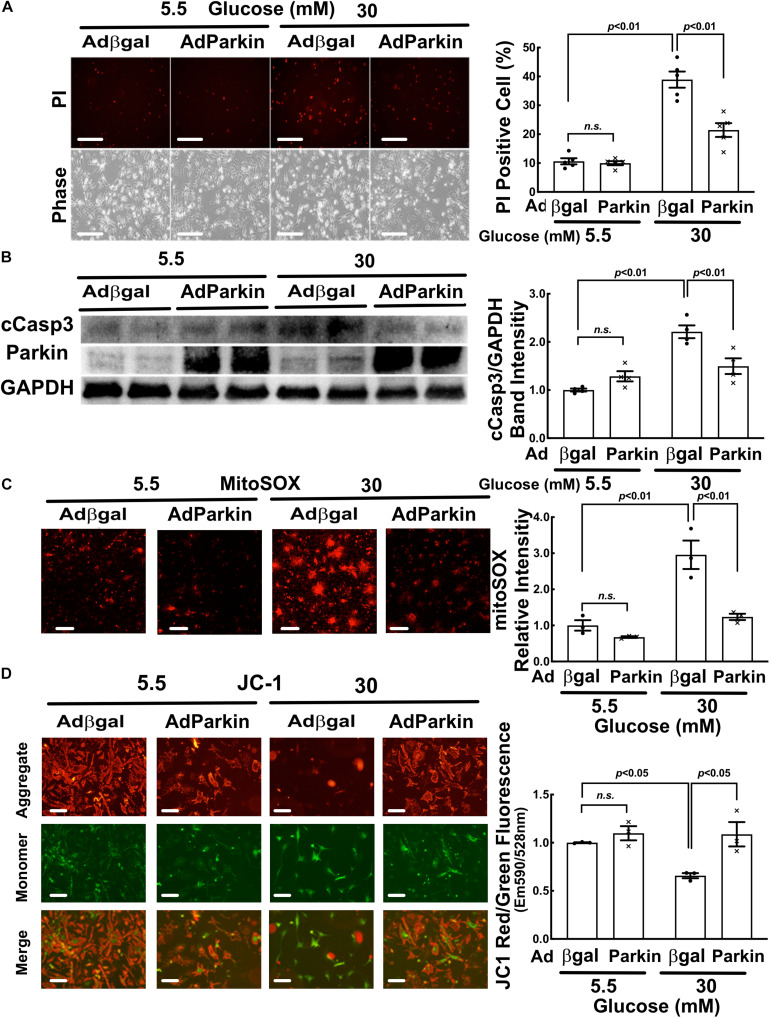
Parkin overexpression diminished high glucose-induced cardiomyocyte injury. NRVCs were infected with AdParkin or Adβgal and cultured in DMEM containing 5.5 or 30 mM glucose for 72 h, and cardiomyocyte injury was determined by the number of PI positive cells (**A:** *p* < 0.01, *n* = 5), the levels of cCasp3 (**B:** *p* < 0.01, *n* = 4), MitoSOX (**C:** *p* < 0.01, *n* = 3) and ΔΨm (**D:** JC-1, *p* < 0.05, *n* = 3). Data were expressed as mean ± SEM and analyzed by two-way ANOVA. Scale bars represent **(A)** 200 μm, **(C)** 200 μm, **(D)** 100 μm, respectively.

**FIGURE 10:**
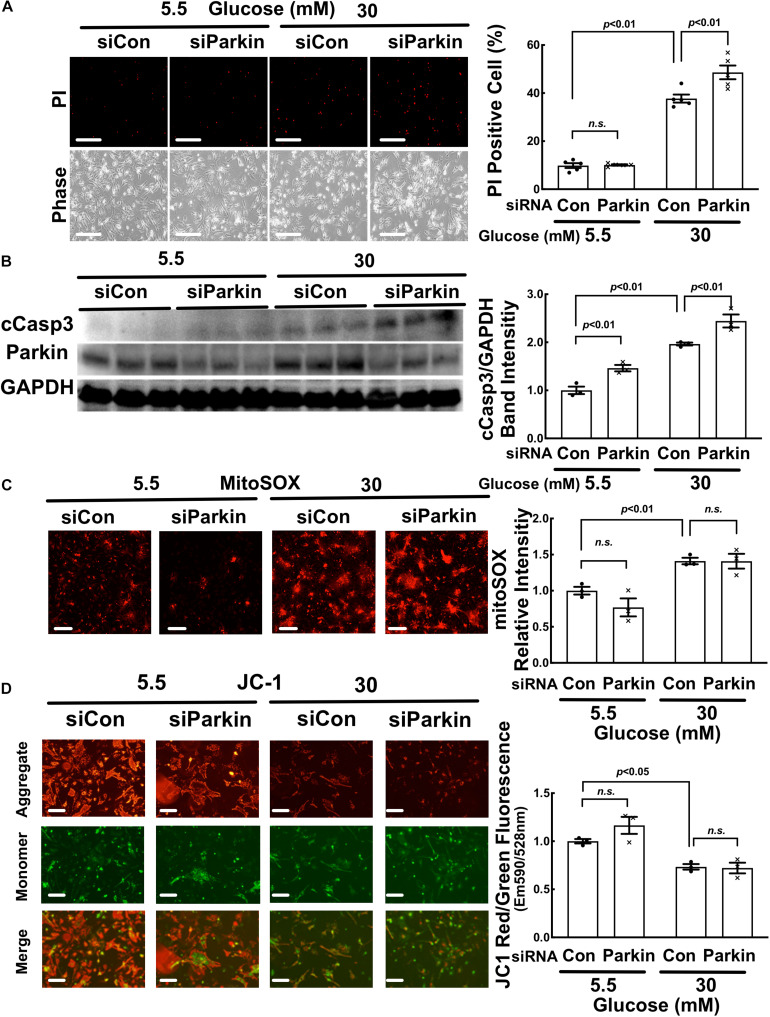
Parkin knockdown exacerbated high glucose-induced cardiomyocyte death. NRVCs were transfected with scrambled control or Parkin-targeted synthetic siRNA, cultured with DMEM containing 5.5 or 30 mM glucose for 72 h, and cardiomyocyte injury was determined by the number of PI positive cells (**A:** *p* < 0.01, *n* = 5), the levels of cCasp3 (**B:** *p* < 0.01, *n* = 3), MitoSOX (**C:** *p* < 0.01, *n* = 3) and ΔΨm (**D:** JC-1, *p* < 0.05, *n* = 3). Data were expressed as mean ± SEM and analyzed by two-way ANOVA. Scale bars represent **(A)** 200 μm, **(C)** 200 μm, **(D)** 100 μm, respectively.

To determine the role of mitophagy in HG-induced cardiomyocyte injury, we overexpressed or silenced Parkin in NRVCs using adenovirus or siRNA knockdown, respectively. Parkin is an E3 ubiquitin ligase that ubiquitinates depolarized or otherwise injured mitochondria to trigger their degradation by mitophagy. Compared to Adβgal-infected NRVCs, the AdParkin moderately increased mitophagy flux under NG conditions, as shown by the difference in the numbers of red dots with and without PepA/E64d (Figures 6A,C). Parkin overexpression also relieved the inhibition of mitophagy flux by HG. Conversely, Parkin knockdown with siRNA inhibited mitophagy flux in NG-cultured cells. It also dramatically decreased the number of red dots in HG-cultured cells either with or without PepA/E64d, although it did not further reduce mitophagy flux (Figure 7A,C). Importantly, increasing mitophagy activity by AdParkin diminished HG-induced cardiomyocyte injury (Figure 9), while Parkin knockdown had the opposite effects (Figure 10), as measured by the number of PI positive cells and the levels of cCasp3, superoxide and ΔΨm. Alternatively, we treated NRVCs with CCCP, a mitochondrial uncoupler that is routinely used to induce mitophagy. We found that a low dose of CCCP (5 nM) protected from HG-induced cardiomyocyte death as shown by the reduced PI positive cells and cCasp3 (Supplementary Figure 4A,B). These results strongly suggested that mitophagy is essential for maintaining mitochondrial health and cardiomyocytes survival in response to HG stress. Interestingly, Parkin overexpression also increased mitochondrial fragmentation as shown by the substantially reduced mitochondrial size (Figures 6A,B), despite the inability of Parkin knockdown to affect mitochondrial fission (Figures 7A,B), suggesting a positive feedback mechanism linking mitophagy and mitochondrial fission.

### High Glucose Induced Mitochondrial Fragmentation but Inhibited Mitophagy in Adult Mouse Cardiomyocytes (AMCs)

**FIGURE 11:**
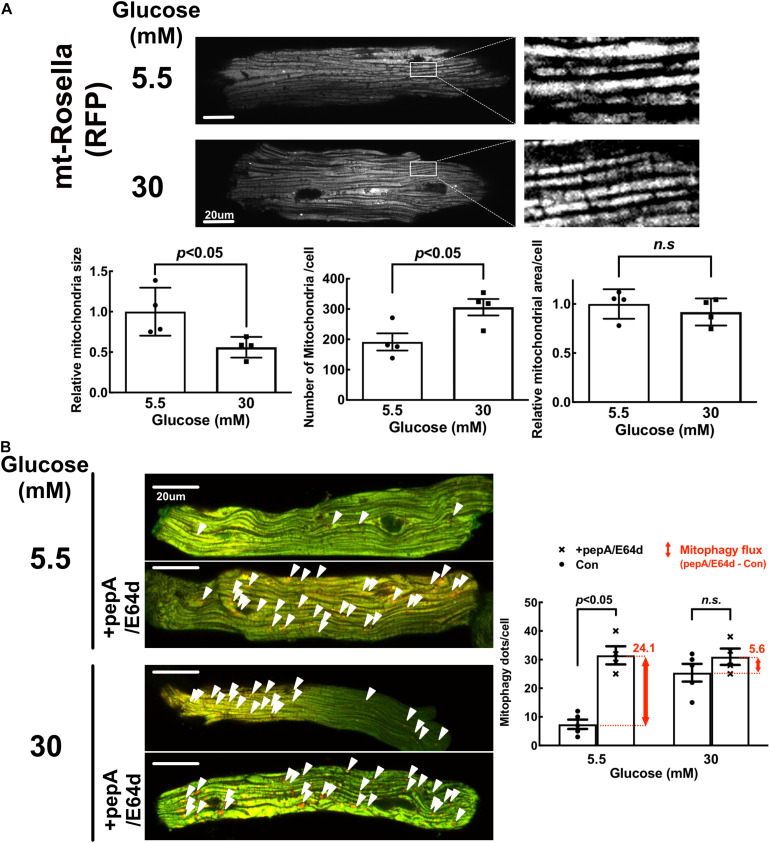
High glucose induced mitochondrial fragmentation but reduced mitophagy flux in adult mouse cardiomyocytes (AMCs). The AMCs were isolated from adult transgenic mouse hearts that express mt-Rosella reporter and cultured with 5.5 or 30 mM glucose for 72 h. **(A)** Red fluorescence images were obtained by confocal microscopy and analyzed with ImageJ. After image optimization, four images were analyzed and the average mitochondrial particle sizes, numbers and areas were calculated. **(B)** The representative images showed mitophagy foci (red puncta indicated by the arrow heads) in AMCs. The number of mitophagy foci was quantified manually. At least four images were captured per treatment. Experiments were repeated with addition of lysosomal inhibitors (PepA and E64D). Mitophagy flux was calculated by subtracting the mean of red puncta without inhibitors from the corresponding mean value of red puncta with inhibitors, which was denoted by the red number and the red vertical line with arrows at both ends in the bar graphs. Data are expressed as mean ± SEM and were analyzed by using unpaired student *t*-test (^∗^*p* < 0.01 vs. 5.5 mM glucose, *n* = 5). Scale bar = 10 μm.

Since the mitochondrial morphology and physiology differ between neonate and adult (Eisner et al., 2017), we performed additional experiments using adult mouse cardiomyocytes (AMCs) isolated from the transgenic mice that express the mt-Rosella mitophagy reporter in the heart. As shown in Figure 11A, HG treatment reduced the mean mitochondrial particle size by approximately 44% (0.56 ± 0.17 compared to NG, *p* < 0.05, *n* = 4), while increased the mitochondrial number by 1.6 fold (1.60 ± 0.2 compared to NG, *p* < 0.05, *n* = 4), suggesting that HG increased mitochondrial fragmentation. However, HG did not have any effect on the total mitochondrial area in AMC (Figure 11A), suggesting that mitochondrial degradation might have been decreased. Indeed, HG increased the number of red dots compared with NG, but treatment with PepA/E64d resulted in a much smaller increase in the number of red dots in cells cultured in HG than in NG (Figure 11B), indicating that HG inhibited mitophagy flux, consistent with the results obtained from NRVCs.

## Discussion

Mitochondrial dysfunction plays a key role in diabetic heart failure. Mitochondrial fission has been observed in the diabetic heart and in cardiomyocytes cultured with high glucose (Liang and Kobayashi, 2016) and has been thought to contribute to glucotoxicity (Yu et al., 2008, 2011, 2017; Makino et al., 2011). However, inhibition of mitochondrial fission reduced mitochondrial respiration (Yu et al., 2006). Also, the mitochondrial uncoupler FCCP that can trigger mitochondrial fragmentation abolished HG-induced cardiomyocyte injury (Yu et al., 2006, 2008), suggesting that mitochondrial fragmentation may not necessarily be detrimental. Indeed, our present study showed that DRP1 knockdown did not reduce HG toxicity (Figure 4) and Mdivi-1, a chemical inhibitor of mitochondrial fission, even increased HG-induced cardiomyocyte death (Supplementary Figure 3). More strikingly, DRP1 overexpression mitigated HG-induced cardiomyocyte injury (Figure 5). These results demonstrated that increased mitochondrial fragmentation did not contribute to HG cardiotoxicity; instead, it protected against HG-induced cardiomyocyte injury, strongly supporting the notion that mitochondrial fragmentation was an adaptive response that limited HG toxicity, in sharp contrast to previous studies (Yu et al., 2008, 2011, 2017; Makino et al., 2011).

Our results are surprising but not entirely unexpected given previous studies that demonstrate a dual role for mitochondrial fission in cardiomyocytes. On one hand, suppressing mitochondrial fission diminishes cardiac damage triggered by a number of different stresses or insults such as ischemia/reperfusion (Ong et al., 2010; Disatnik et al., 2013; Sharp et al., 2014), pressure overload (Givvimani et al., 2012) and doxorubicin (Gharanei et al., 2013; Catanzaro et al., 2019), suggesting a detrimental role for mitochondrial fission. On the other hand, a DRP1 loss-of-function mutation (Ashrafian et al., 2010; Cahill et al., 2015) or cardiac specific inactivation of DRP1 (Kageyama et al., 2014; Ikeda et al., 2015; Ishihara et al., 2015; Song et al., 2015a,b) invariably leads to heart failure, underscoring an essential role of mitochondrial fission in maintaining cardiac homeostasis. A protective role of mitochondrial fission has also been shown in cultured cardiomyocytes (Brand et al., 2018). Together, these studies reveal the dichotomous nature of mitochondrial fragmentation that can either protect or damage the heart under different conditions. Then, what determines the ultimate effects of mitochondrial fragmentation on cardiomyocytes? One possibility may have something to do with the functional state of mitophagy. Mitochondrial fission separates injured mitochondrial fragments and channels them into mitophagic process for degradation (Twig et al., 2008). If mitophagy can efficiently eliminate these fragments, the whole process will be cardioprotective. However, if mitophagy is impaired or fission is overwhelming, the fragmented dysfunctional mitochondria will accumulate and cause cardiac injury (Liang and Kobayashi, 2016).

Using a novel dual fluorescent mitophagy reporter known as mt-Rosella, we showed that HG increased mitophagy foci (red dots) in both neonatal and adult cardiomyocytes, suggesting that HG might have enhanced mitophagy. However, treatment with lysosomal degradation inhibitors resulted in a smaller increase in the number of red dots in cells cultured in HG, indicating that HG actually reduced mitophagy flux (Figures 3A,C, 4A,C, 6A,C,7A,C, 11B). A major positive regulator of mitophagy is Parkin, an E3 ligase that adds ubiquitin tag to the target proteins on the damaged mitochondria, promoting mitophagosome formation. The expression levels of Parkin did not appear to be altered by HG treatment (Figures 9B, 10B and Supplementary Figure 2A), which seemed contradictory to the fact that HG reduced mitophagy flux. However, the Parkin-mediated formation of mitophagosome is only the first step in the entire mitophagy process. If the degradation is insufficient, the mitophagy process cannot be carried out to the completion. Indeed, as we showed recently (Kobayashi et al., 2020b), HG was able to induce lysosomal membrane permeabilization (LMP), which might compromise lysosomal function, impeding mitophagic degradation. It is thus possible that mitophagy flux may be limited to some degree even if Parkin-mediated mitophagosome formation remains relatively normal under HG conditions. Nevertheless, overexpression of Parkin, a positive regulator of mitophagy, was able to not only accelerate mitophagy (Figure 6C) but also attenuate HG-induced cardiomyocyte death (Figure 9). These results suggest that enhancing the initiation of mitophagy, i.e., the formation of mitophagosomes, could still be a promising strategy for reducing hyperglycemic cardiotoxicity if the downstream lysosomal function is not severely impaired.

Of note, Parkin overexpression not only enhanced mitophagy but also increased mitochondrial fragmentation (Figure 6B), consistent with two previous studies showing that BCL2L13, a mitophagy receptor, was able to concurrently induce mitophagy and mitochondrial fragmentation in HEK293 cells (Dagda et al., 2009; Brand et al., 2018). These results demonstrated a feed-back activation of the fission process by mitophagy which may generate more fragmented mitochondria further stimulating mitophagy as we suggested before (Liang and Kobayashi, 2016). However, other studies showed that Parkin inhibited mitochondrial fragmentation in SH-SY5Y cells (Dagda et al., 2009; Lutz et al., 2009; Wang et al., 2011), exhibiting a feed-back inhibition of fission by mitophagy, in contrast to the above results, which may serve as a brake to prevent excessive mitophagy in this specific context. Nevertheless, it remains to be determined if this feed-back inhibition mechanism exists in cardiomyocytes under conditions other than HG.

**FIGURE 12:**
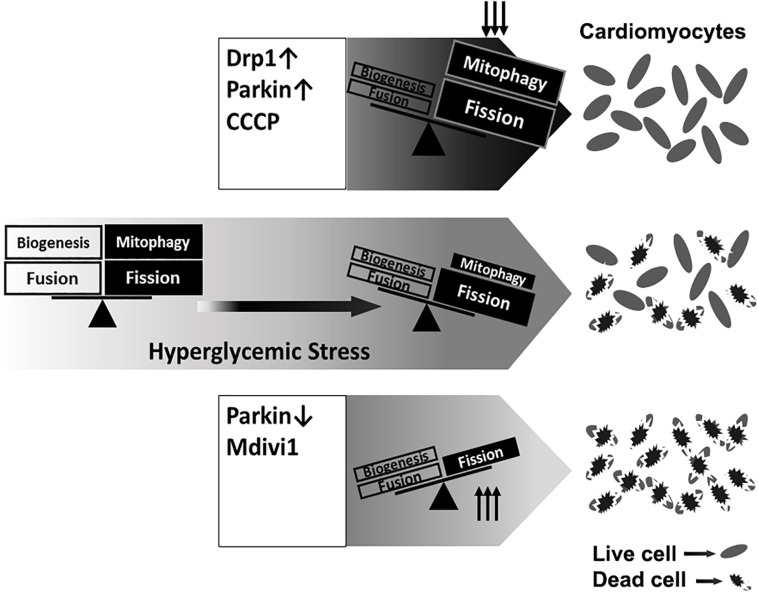
A graphic presentation of the roles of mitochondrial fission and mitophagy in high glucose cardiotoxicity. Mitochondrial quality is controlled by a number of coordinated mechanisms including mitochondrial biogenesis, fission and mitophagy. Injured mitochondrial segments are separated by mitochondrial fission and eliminated by mitophagy. High glucose (hyperglycemic stress) induced mitochondrial fragmentation and inhibited mitophagy, which was associated with increased cardiomyocyte death. Overexpression of DRP1 and Parkin or treatment with the mitochondrial uncoupler CCCP each increased mitophagy and protected from high glucose-induced cardiomyocytes death. Conversely, the fission inhibitor Mdivi-1 and Parkin knockdown exacerbated high glucose-induced cardiomyocyte death. These results suggest that promoting the positive interaction between mitochondrial fission and mitophagy while simultaneously maintain their levels within the physiological range would be expected to improve mitochondrial health, alleviating hyperglycemic cardiotoxicity.

Interestingly but not surprisingly, DRP1 overexpression not only increased mitochondrial fission, but also accelerated mitophagy flux (Figure 4C), which may partly account for the protective effects of DRP1-dependent mitochondrial fission. Collectively, these data revealed a reciprocal positive feedback/forward loop that controls mitochondrial fission and mitophagy in cardiomyocytes (Figure 12). Apparently, further studies are needed to elucidate the underlying signaling mechanisms that regulate the interaction or cross-talk between mitochondrial fission and mitophagy. This may lead to the identification of new therapeutic strategies to reducing hyperglycemic cardiotoxicity.

In summary, the present study showed that HG-induced mitochondrial fragmentation was an adaptive response that served to limit rather than mediate HG cardiotoxicity as previously thought. We also revealed a positive regulatory loop by which mitochondrial fission and mitophagy activated each other to enhance mitochondrial quality control. Accordingly, strategies that promote the reciprocal positive interaction between mitochondrial fission and mitophagy and simultaneously keep their levels within the physiological range would be expected to improve mitochondrial health, alleviating HG cardiotoxicity.

## Data Availability Statement

The original contributions presented in the study are included in the article/Supplementary Material, further inquiries can be directed to the corresponding author/s.

## Ethics Statement

The animal study was reviewed and approved by the Institutional Animal Care and Use Committee at NYIT College of Osteopathic Medicine.

## Author Contributions

SK and QL designed the research, analyzed the data, and wrote the manuscript. SK, FZ, ZZ, TK, and YH performed the research. BS and WW edited the manuscript. All authors contributed to the article and approved the submitted version.

## Conflict of Interest

The authors declare that the research was conducted in the absence of any commercial or financial relationships that could be construed as a potential conflict of interest.

## Abbreviations

Fig: Figure
NG: normal glucose (5.5 mM)
HG: High glucose (30 mM)
ROS: reactive oxygen species
cCasp3: cleaved Caspase 3
PARP: Poly (ADP-ribose) polymerase
DMEM: Dulbecco’s modified Eagle medium
DMSO: dimethyl sulfoxide
GAPDH: glyceraldehyde-3-phosphate dehydrogenase
GFP: green fluorescent protein
LAMP-1: lysosomal-associated membrane protein 1
RFP: red fluorescent protein
PGC-1 α: Peroxisome proliferator-activated receptor gamma coactivator 1-alpha
COX IV: Cytochrome c oxidase subunit 4
Mfn1/2: Mitofusin 1/2
Opa1: optic atrophy 1
PINK1: PTEN-induced kinase 1
DRP1: dynamin-related protein 1
PepA: Pepstatin A
PI: Propidium iodide
mdivi-1: mitochondrial division inhibitor 1
CCCP: carbonyl cyanide m-chlorophenylhydrazone
siRNA: short interference RNA
β -gal: β -galactosidase
LC3: microtubule-associated protein Light Chain 3
Supp: supplemental.
