# Supplementary material for: Mitochondrial Fission and Mitophagy Coordinately Restrict High Glucose Toxicity in Cardiomyocytes
Source: Front Physiol. 2020 Dec 10;11:604069. doi: 10.3389/fphys.2020.604069 (PMC7758327; doi:10.3389/fphys.2020.604069)

Supplemental Figure Legend

Supplemental Figure 1. A time course of **high glucose induced mitochondrial fragmentation.** NRVCs were infected with Ad-mt-Rosella and cultured in DMEM with 30mM glucose for various time periods (3 to 72 hours). Mitochondrial structure was visualized by GFP of mt-Rosella under a confocal microscope (A). The scale bars=20μm. The mean values of mitochondrial sizes and the numbers per cell were plotted against the time (B). Data were expressed as mean ± SEM and were analyzed by using one-way ANOVA (*$p<0.01$ (B) or <0.05 (C) vs. 5.5 mM glucose (0 hour), N = 3-5).

Supplemental Figure 2. The effects of high glucose on mitochondrial fission/fusion/mitophagy proteins and mitochondrial DNA (mtDNA) in cardiomyocytes. NRVCs were cultured in DMEM with 5.5mM or 30mM glucose for 72 hours. A. Western blot analyses of the protein expression levels in total lysates (Total) and mitochondrial fractions (Mito). B. The mitochondrial DNA (mtDNA) copy number was measured by quantitative PCR. mtDNA levels were calculated as a ratio of mtDNA-encoded target gene COX IV to the nuclear DNA-encoded reference gene GAPDH. Each value is shown as the mean ± SEM of three replicates.

Supplemental Figure 3. Mitochondrial division inhibitor 1 (mdivi-1) exacerbated high glucose-induced cardiomyocyte death. NRVCs were cultured in DMEM containing 5.5 or 30 mM glucose and treated with mdivi-1 (1 μM) for 72 hours. Cardiomyocyte death was evaluated by PI positive cells (A) and cCasp3 (B). Data are expressed as mean ± SEM and were analyzed by two-way ANOVA ($p<0.01$, n=3).

Supplemental Figure 4. CCCP protected from HG-induced cardiomyocyte death. NRVCs were cultured in DMEM containing 5.5 or 30 mM glucose and treated with CCCP (5 nM) for 72 hours. Cardiomyocyte death was evaluated by PI positive cells (A) and cCasp3 (B). Data are expressed as mean ± SEM and were analyzed by two-way ANOVA ($p<0.01$, n=3).

Suppl. Figure 1

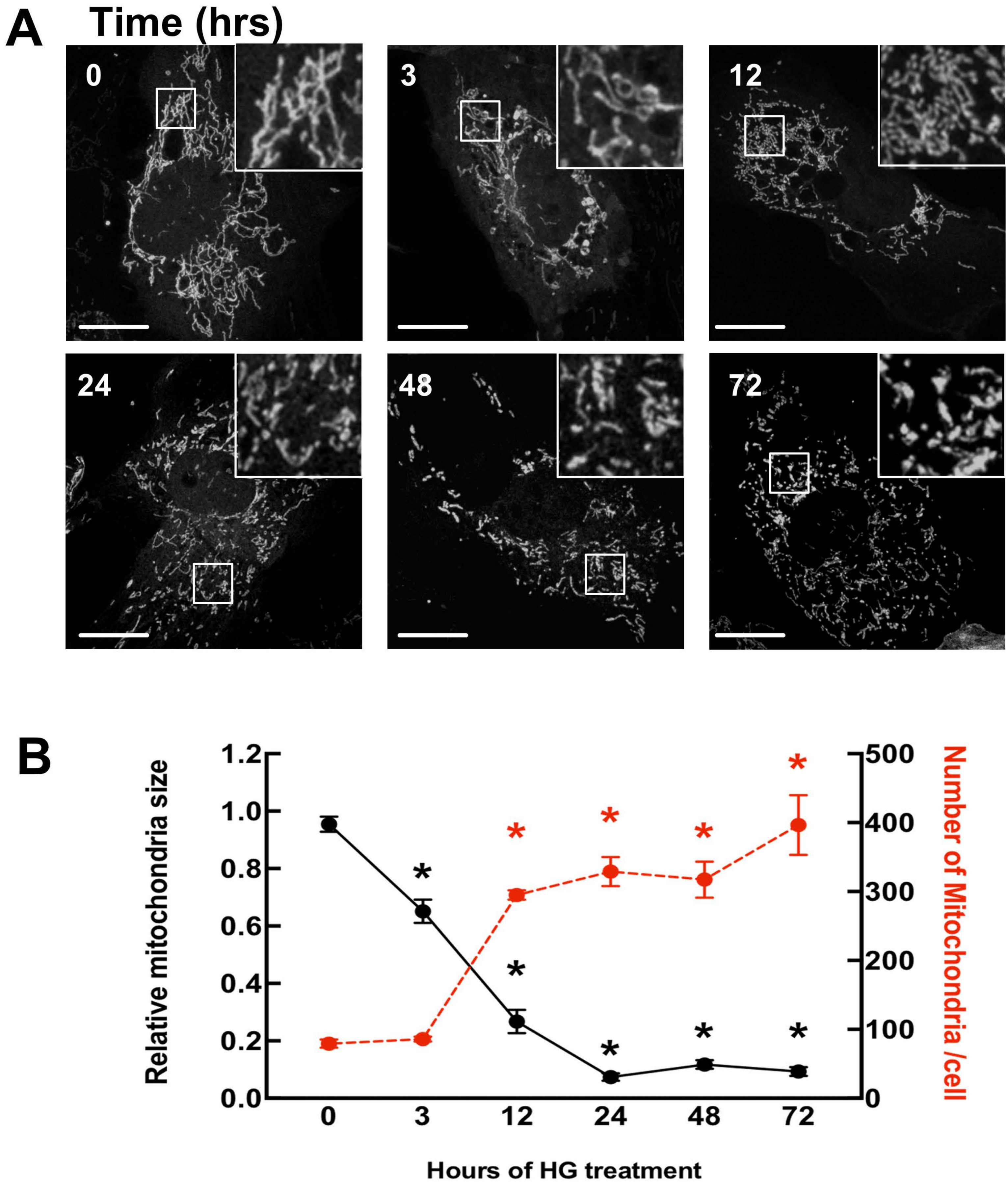

Suppl. Figure 2

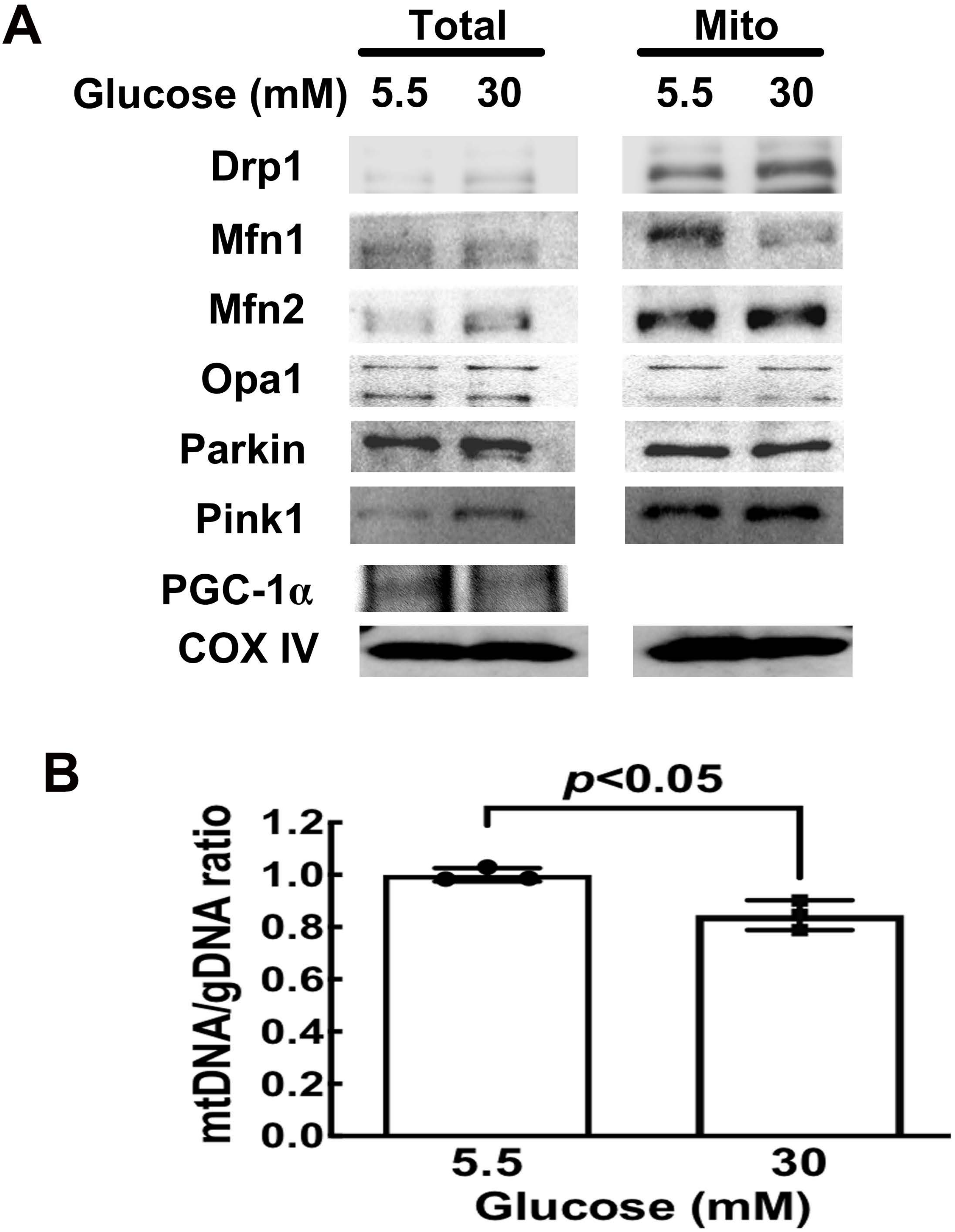

Supplementary Figure 3

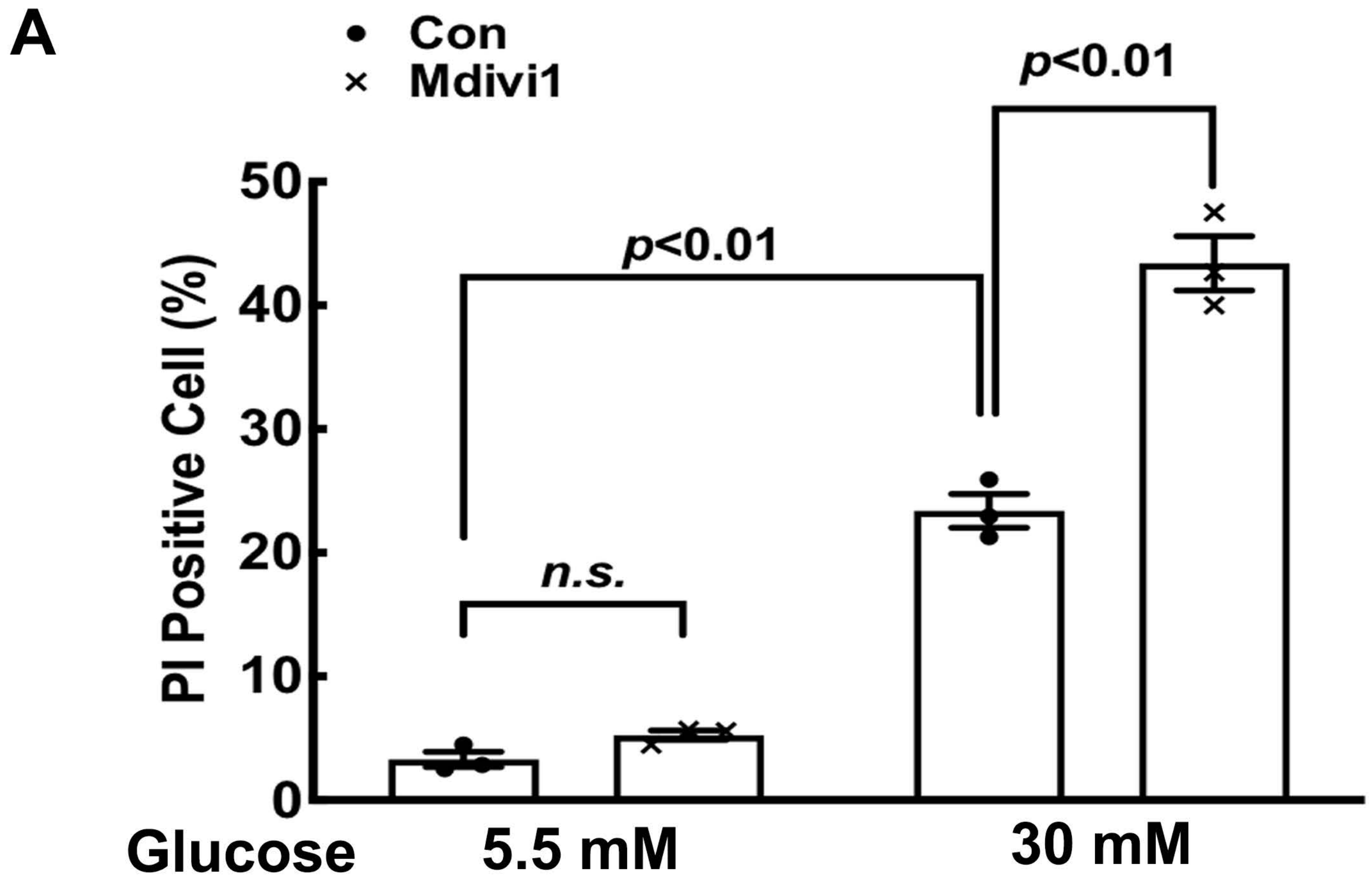

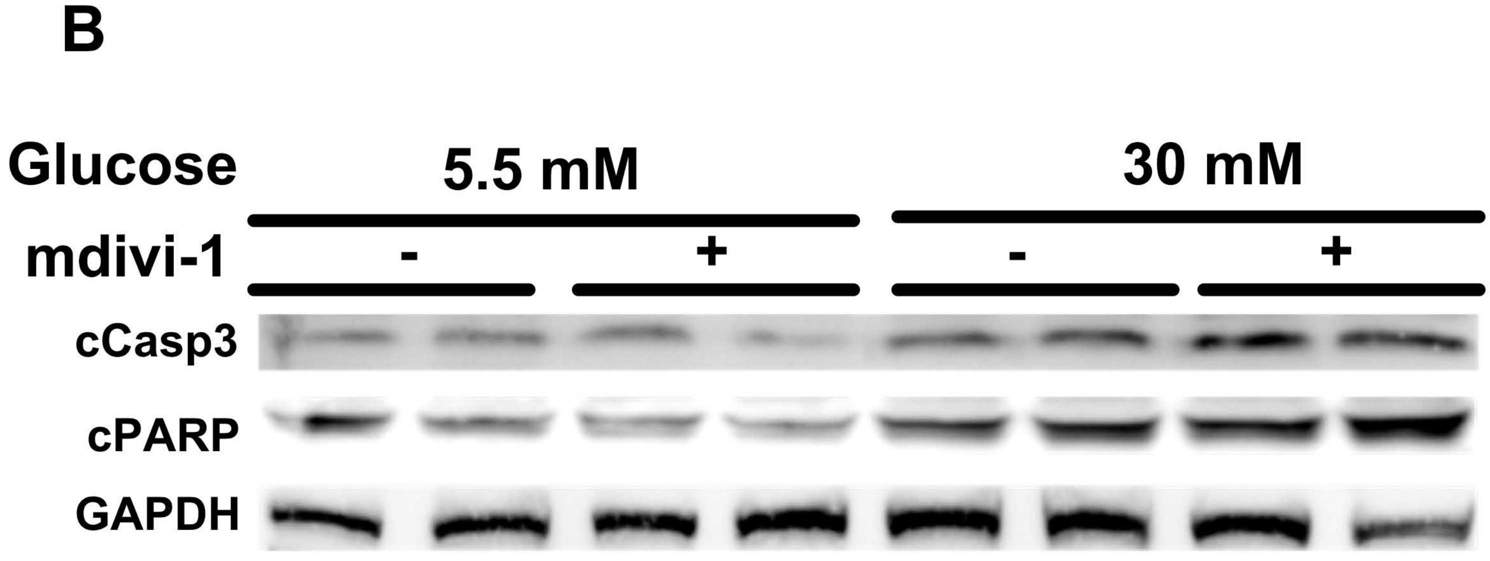

Supplementary Figure 4

A

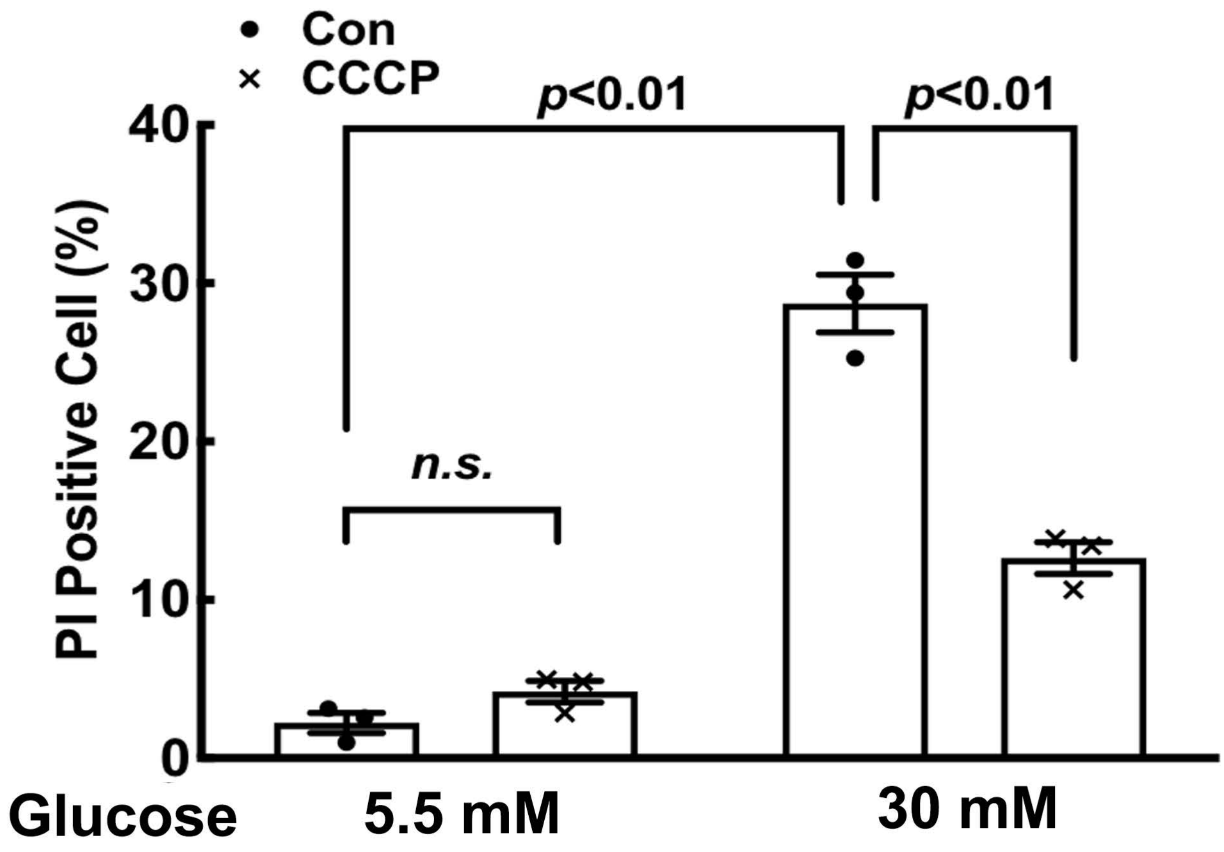

B

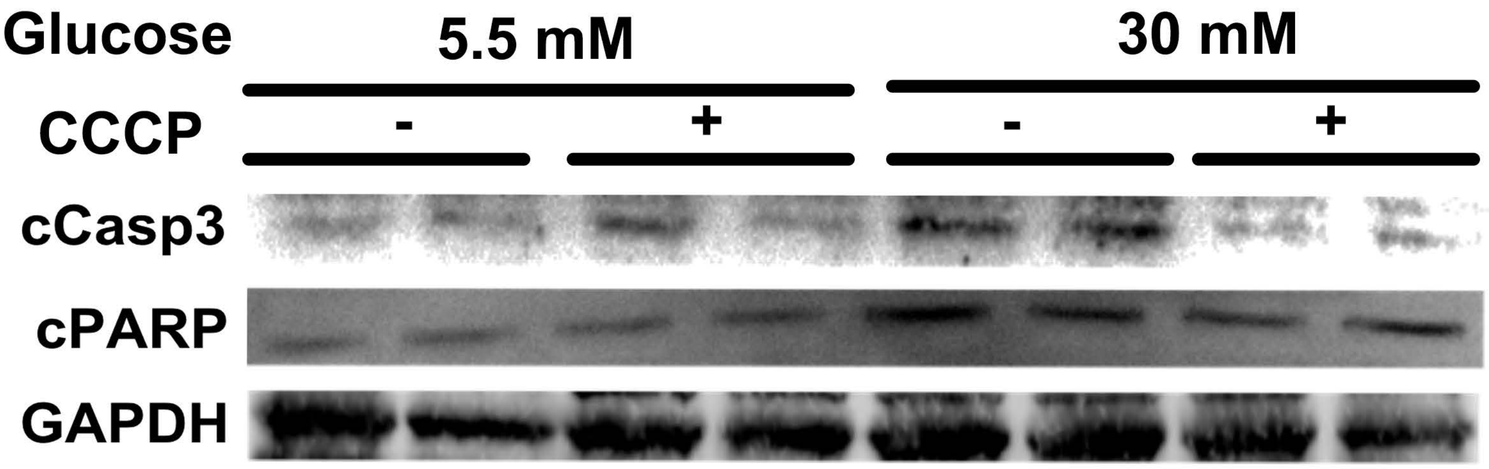

Supplement: Supplementary file 1 [file Data_Sheet_1.pdf]
